# A 50 Hz magnetic field influences the viability of breast cancer cells 96 h after exposure

**DOI:** 10.1007/s11033-022-08069-7

**Published:** 2022-11-15

**Authors:** Maria Elexpuru-Zabaleta, Raffaella Lazzarini, Maria Fiorella Tartaglione, Francesco Piva, Veronica Ciarapica, Elena Marinelli Busilacchi, Antonella Poloni, Matteo Valentino, Lory Santarelli, Massimo Bracci

**Affiliations:** 1 Research Group on Foods, Nutritional Biochemistry and Health, Universidad Europea del Atlántico, 39011 Santander, Spain; 2 Occupational Medicine, Department of Clinical and Molecular Sciences, Polytechnic University of Marche, 60126 Ancona, Italy; 3 Department of Specialistic Clinical and Odontostomatological Sciences, Polytechnic University of Marche, 60131 Ancona, Italy; 4 Section of Hematology, Department of Clinical and Molecular Science, Polytechnic University of Marche, 60126 Ancona, Italy

**Keywords:** Extremely low frequency magnetic field, Breast cancer, Cell viability, MDA-MB-231, MCF-7, MCF-10A

## Abstract

**Background:**

The exposure of breast cancer to extremely low frequency magnetic fields (ELF-MFs) results in various biological responses. Some studies have suggested a possible cancer-enhancing effect, while others showed a possible therapeutic role. This study investigated the effects of in vitro exposure to 50 Hz ELF-MF for up to 24 h on the viability and cellular response of MDA-MB-231 and MCF-7 breast cancer cell lines and MCF-10A breast cell line.

**Methods and results:**

The breast cell lines were exposed to 50 Hz ELF-MF at flux densities of 0.1 mT and 1.0 mT and were examined 96 h after the beginning of ELF-MF exposure. The duration of 50 Hz ELF-MF exposure influenced the cell viability and proliferation of both the tumor and nontumorigenic breast cell lines. In particular, short-term exposure (4–8 h, 0.1 mT and 1.0 mT) led to an increase in viability in breast cancer cells, while long and high exposure (24 h, 1.0 mT) led to a decrease in viability and proliferation in all cell lines. Cancer and normal breast cells exhibited different responses to ELF-MF. Mitochondrial membrane potential and reactive oxygen species (ROS) production were altered after ELF-MF exposure, suggesting that the mitochondria are a probable target of ELF-MF in breast cells.

**Conclusions:**

The viability of breast cells in vitro is influenced by ELF-MF exposure at magnetic flux densities compatible with the limits for the general population and for workplace exposures. The effects are apparent after 96 h and are related to the ELF-MF exposure time.

**Supplementary Information:**

The online version contains supplementary material available at 10.1007/s11033-022-08069-7.

## Introduction

During recent decades, scientists have aimed to elucidate how extremely low frequency (ELF) electromagnetic fields, ubiquitous in modern society, affect living organisms, including humans [1–3]. The use of electric devices and equipment in clinical practice, industrial environments, and common domestic situations generates ELF magnetic fields (ELF-MF) with frequencies of 50–60 Hz [1, 4, 5].

Several epidemiologic surveys and in vivo/in vitro biological studies have focused on the possible adverse health effects that may be associated with ELF-MF [1, 6, 7]. In particular, the correlation between ELF-MF exposure and cancer risk has become a matter of public concern. Moreover, in 2002, the International Agency for Research on Cancer (IARC) classified ELF-MF as a possible carcinogen in humans (Group 2B) [8]. Considering the growing evidence from the scientific community of interactions between ELF-MF and biological systems, laws have been made to establish exposure limits for the population. On the other hand, the exposure limits for ELF-MF in workplaces are commonly higher than those for the general population. The current exposure limit for 50 Hz ELF-MF in the general population is 0.1 mT according to the European Union Recommendation 1999/519/EC [9]. For workers, the low action limit is 1.0 mT, but higher exposures are possible according to the European Directive 2013/35/EU [10, 11]. Several international organizations have suggested different limits for ELF-MF. The International Commission on Non-Ionizing Radiation Protection (ICNIRP) guidelines indicated 50 Hz ELF-MF exposures reference limits of 100 μT for the general public and 500 μT for workers [12]. The Institute of Electrical and Electronics Engineers (IEEE) recommends 50 Hz EMF-MF exposure limit levels of 904 μT for the general public and 2710 μT for workers [13].

Although these exposure limits were established as a precautionary measure, scientific studies have highlighted some biological effects of ELF-MF with magnetic flux density within the legally permitted range [6, 14–16]. In vitro studies on cancer cell proliferation induced by ELF-MF report conflicting findings [1, 8, 17]. Some researchers have shown an augmentation of malignant and/or normal cell proliferation after exposure to ELF-MF. In this respect, Wolf and collaborators have shown that exposure of HL-60 leukemia cells and rat fibroblasts to 50 Hz (0.5–1.0 mT) ELF-MF influenced proliferation and DNA damage through the action of free radical species in a dose- and time-dependent manner [3]. In addition, Falone and collaborators reported that 50 Hz (1.0 mT) ELF-MF provides a survival advantage to cancer cells through the activation of the antioxidative and detoxification defense systems, conferring significant drug resistance to cells [4]. There is also a growing interest in using electromagnetic fields as a medical or anticancer treatment. ELF-MF have been demonstrated to accelerate wound healing, enhance musculoskeletal recovery, and disrupt tumor growth [18–20]. Some types of malignant cells are particularly vulnerable to the effects of ELF-MF, as it influences the mechanisms regulating cell proliferation [21, 22].

Worldwide, breast cancer is the most commonly diagnosed cancer [23]. Although the epidemiological evidence of an association between breast cancer and exposure to ELF-MF is not consistent, several studies highlight an increase in risk [24]. Scientific data on ELF-MF and breast cancer in animal models are not clear but suggest a possible cancer-promoting effect in combination with other initiating agents (e.g., γ-rays) [25]. Studies conducted on breast cells have shown the effects of EMF-MF on physiological functions and protein expression [26–29] and a possible therapeutic role of treatment with ELF-MF in combination with chemotherapy in breast cancer was suggested [22, 30]. However, conclusions are still controversial.

This study investigated the effect of exposure to 50 Hz ELF-MF for up to 24 h on the viability and proliferation of three breast cell lines. Two ELF-MF flux densities of 0.1 mT (limit for the general population) and 1.0 mT (magnetic flux density permitted in workplaces) were tested.

## Materials and methods

### Cell lines

MDA-MB-231 (triple-negative human breast cancer cell line), MCF-7 (estrogen-receptor-positive human breast cancer cell line), and MCF-10A (human breast epithelial cell line) were acquired from the Experimental Zooprophylactic Institute of Lombardia and Emilia Romagna (Brescia, Italy). MDA-MB-231 and MCF-7 cells were cultured in Dulbecco’s modified Eagle’s medium (DMEM; Euroclone, Pero, Italy) supplemented with 10% fetal bovine serum (FBS), 100 IU/mL penicillin (Euroclone), and 100 g/L streptomycin (Euroclone). MCF10A cells were cultured in DMEM/F12 medium with L-glutamine (Euroclone) supplemented with 5% heat-inactivated horse serum (Euroclone), 20 ng/ml epidermal growth factor (Merck Life Science, Milan, Italy), 0.5 μg/ml hydrocortisone (Merck Life Science), 100 ng/ml cholera toxin (Merck Life Science), 10 µg/ml insulin (Merck Life Science), and penicillin (100 U/ml)/streptomycin (100 μg/ml) (Euroclone). All three cell lines were kept in a humidified 5% CO_2_-air atmosphere at 37 °C and split twice a week.

### Electromagnetic field exposure

A square-shaped Helmholtz coil and an electrical current generator were used as the exposure system. The system was self-designed and built to generate a 50 Hz ELF-MF suitable for cell exposure inside a cell incubator. Considering the thermal insulation of a cell incubator, the Helmholtz coil was designed to generate low temperatures. The thickness of the copper wire of the coil was chosen to keep the electric power dissipated within 8 Watts. The length of the inner side of the coil was 34 cm. The ELF-MF produced by our generator was verified with a professional ELF-MF analyzer (EFA-300; Wandel & Goltermann, Germany). Cells exposed to 50 Hz ELF-MF (0.1 and 1.0 mT) were placed after seeding (0 h) at the center of the Helmholtz coil positioned into a temperature- and atmosphere-regulated incubator (37.0 ± 0.1 °C and 5% CO_2_). The control group was placed in another incubator and subjected to the same procedures as experimental cells but without 50 Hz ELF-MF exposure. After the 50 Hz ELF-MF exposure, the exposed cells were quickly transferred to the other incubator (near the control group) until analysis at 48, 96 and 192 h after seeding. To exclude any thermal effects, the temperature at the cell level of both groups was constantly monitored during experiments with a Thermochron iButton DS1922L (Maxim Integrated, San Jose, CA, USA).

### Cell viability assay

To assess the effect of 50 Hz ELF-MF on cell viability after 48 and 96 h, the XTT assay was performed following the manufacturer’s guidelines (Cell Proliferation Kit II XTT; Merck Life Science). Briefly, tumor and normal cells were seeded in 96-well plates (Costar, Corning Incorporated, Corning, NY, USA) and immediately exposed to 50 Hz ELF-MF for different duration. At 48, 96 and 192 h after seeding, 75 µL of the XTT (2,3-bis-(2-methoxy-4-nitro-5-sulfophenyl)-2H-tetrazolium-5-carboxanilide) solution was added to each well (150 µL), followed by incubation for 4 h at 37 °C and 5% CO_2_. Finally, the absorbance at 450 nm with 650 nm as the reference wavelength was measured using an ELISA microplate absorbance reader (Sunrise; Tecan Group Ltd., Männedorf, Switzerland).

### Trypan blue assay

The trypan blue assay was performed 96 and 192 h after the beginning of ELF-MF exposure. Cells were detached, centrifuged at 800 rpm for 5 min, resuspended in 1 ml of culture medium, and incubated in 0.4% trypan blue at 1:1 (Merck Life Science). A homogeneous suspension of cells was then deposited into a Burker chamber (Merck Life Science) and counted. The number of total live cells was calculated as a percentage of the control.

### Cell cycle analysis

Analysis of cell cycle distribution was performed 96 h after seeding. Cellular DNA content was determined by fluorescence measurement of propidium iodide (PI)-stained cells on a linear scale in a FACSCalibur (BD Biosciences) instrument equipped with Cell Quest software (BD Pharmingen). After harvesting, cells were fixed in 70% ethanol for 2 h and then rinsed with phosphate-buffered saline (PBS) and finally incubated in a solution containing 20 ng/ml RNase A (Merck Life Science) and 5 µg/ml PI (Merck Life Science) for 15 min at 37 °C in the dark. The fluorescence of a minimum of 20,000 cells was acquired for each sample, and the percentage of cells in the different stages of the cell cycle (G_0_/G_1_, S, and G_2_/M phases) was analyzed with FlowJo 7.6.1 software (Tree Star Inc., OR, USA).

### Measurement of intracellular reactive oxygen species levels

Intracellular reactive oxygen species (ROS) levels were detected using the fluorogenic probe 2,7-dichlorofluorescein diacetate (DCFH-DA, Merck Life Science). Cells (10^4^ cells per well) were added to dark 96-well microplates and incubated at 37 °C in 5% CO_2_ for 24 h. Cells were exposed to each experimental condition. Cells were stained with DCFH-DA (15 µM) at 37 °C for 30 min and then washed with 1 × warm PBS. The cellular fluorescence was immediately measured by a fluorescence microplate reader (Tecan Infinite F200, Tecan Group Ltd., Männedorf, Switzerland). Cellular autofluorescence was subtracted, and the fluorescence intensity was expressed relative to the signal of the control.

### Measurement of mitochondrial membrane potential (ΔΨ_M_) using JC-1 dye

Cells were cultured in 96-well black microtiter plates and exposed to ELF-MF for 4 or 24 h. After 96 h, the cell culture medium was replaced with 100 µL/well lipophilic cation 5,5′,6,6′-tetrachloro-1,1′,3,3′-tetraethylbenzimidazolcarbocyanine iodide (JC-1) dye solution (Merck Life Science) at a final concentration of 2 µM and incubated (5% CO_2_, 37 °C) for 20 min in the dark. Next, fluorescence at Ex/Em 535 nm/595 nm for JC1 aggregates and Ex/Em 485 nm/535 nm for JC-1 monomers was measured using an ELISA microplate absorbance reader (Sunrise; Tecan Group Ltd). The value of mitochondrial potential was calculated as the ratio of aggregate to monomer fluorescence values. Finally, values were corrected for protein concentration quantified using a Bradford assay (Merck Life Science).

### Statistical analysis

Experiments were conducted in triplicate, and the results are reported as the mean ± SD of three independent experiments. At the time of each assay, the cell density always remained below confluence, which minimized the potential contributions of cell density-induced changes in biochemical status or nutrient deprivation to the measurements that were performed. GraphPad Prism software (version 7.00 for Windows; GraphPad Software, Inc.) was used for statistical analyses. One-way ANOVA and Dunnett’s post hoc test were used to evaluate statistical significance of differences among groups. Student’s t test was used to test differences in independent measures between two groups. Differences were considered statistically significant when p < 0.05.

## Results

### The duration of 50 Hz ELF-MF exposure influenced the cell viability of tumor and nontumorigenic breast cell lines 96 h after treatment

To analyze the effect of ELF-MF exposure on cell viability over time, we exposed breast cells to 50 Hz 0.1 mT or 1.0 mT ELF-MF for different durations, up to 24 h. Unexposed cells were used as a control and cultured in a normal incubator. The cell viability of each cell line was quantified relative to that of the corresponding unexposed cells. Cells were analyzed after both 48 h and 96 h of incubation.

After 48 h, cell viability did not show differences between exposed (0.1 mT or 1.0 mT ELF-MF) and unexposed cells for all three breast cell lines (data not shown).

**Fig. 1:**
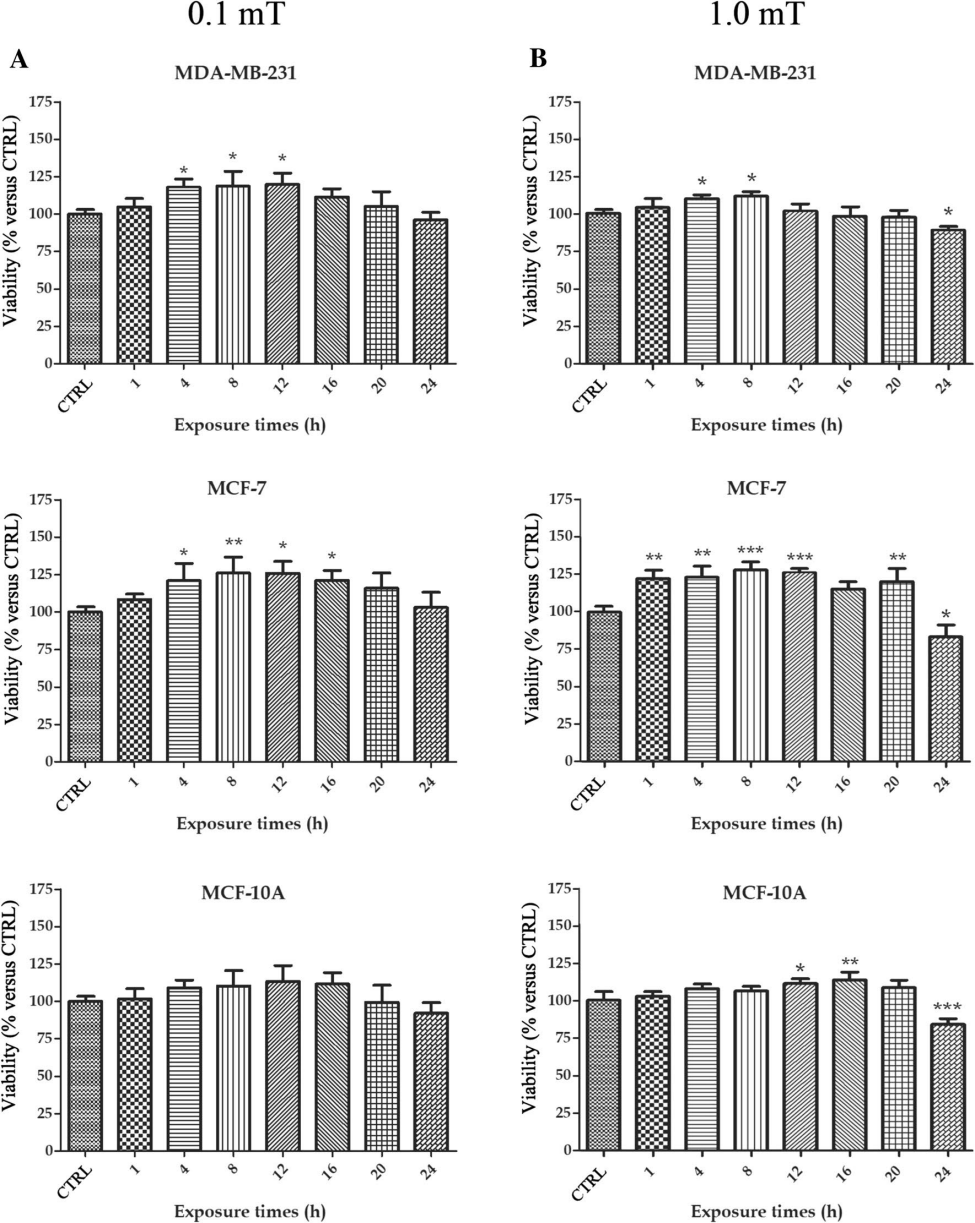
Cell viability after 96 h from the 50 Hz ELF-MF exposure at 0.1 and 1.0 mT of MB-MDA-231, MCF-7 and MCF-10A cell lines. Cells were exposed to 50 Hz 0.1 mT ELF-MF (**A**) or 1.0 mT ELF-MF (**B**) for 1, 2, 4, 8, 12, 16, 20, and 24 h to analyze through an XTT assay the viability during time. Results were analyzed at 96 h after start of treatment. Values for the exposed cells were expressed as the percentage with respect to the not exposed group. Data show mean ± SD. *p < 0.05, **p < 0.01 and ***p < 0.001 vs. CTRL (0 h)

After 96 h, an increase in cell viability in breast cancer cell lines treated with 0.1 mT ELF-MF was observed (Fig. 1A). Specifically, MDA-MB-231 cell viability increased after an exposure time ranging from 4 to 12 h (Fig. 1A), while a noticeable increase in cell viability was observed in MCF-7 cells exposed to 0.1 mT ELF-MF from 4 to 16 h (Fig. 1A).

Similarly, exposure to 1.0 mT ELF-MF for 4 and 8 h increased the viability of MDA-MB-231 cells, while exposure for 24 h decreased cell viability (Fig. 1B). The cell viability increased in MCF-7 cells exposed for 1, 4, 8, 12, and 20 h and decreased in these cells exposed for 24 h (Fig. 1B). In MCF-10A cells, 12 and 16 h of exposure to 1.0 mT ELF-MF increased cell viability, while 24 h exposure decreased viability in these cells (Fig. 1B).

To investigate the effects of ELF-MF exposure on the long-term viability of breast cells, the three cell lines were analyzed at 192 h after the start of 4 h of exposure to 0.1 mT or 1.0 mT ELF-MF (Supplementary file Fig. 1). After 192 h, no significant differences in the viability of any of the three breast cell lines were found between 0.1 or 1.0 mT ELF-MF-treated and control cells.

### Exposure to 50 Hz ELF-MF altered the proliferation of tumor breast cancer cell lines 96 h after ELF-MF treatment

**Fig. 2:**
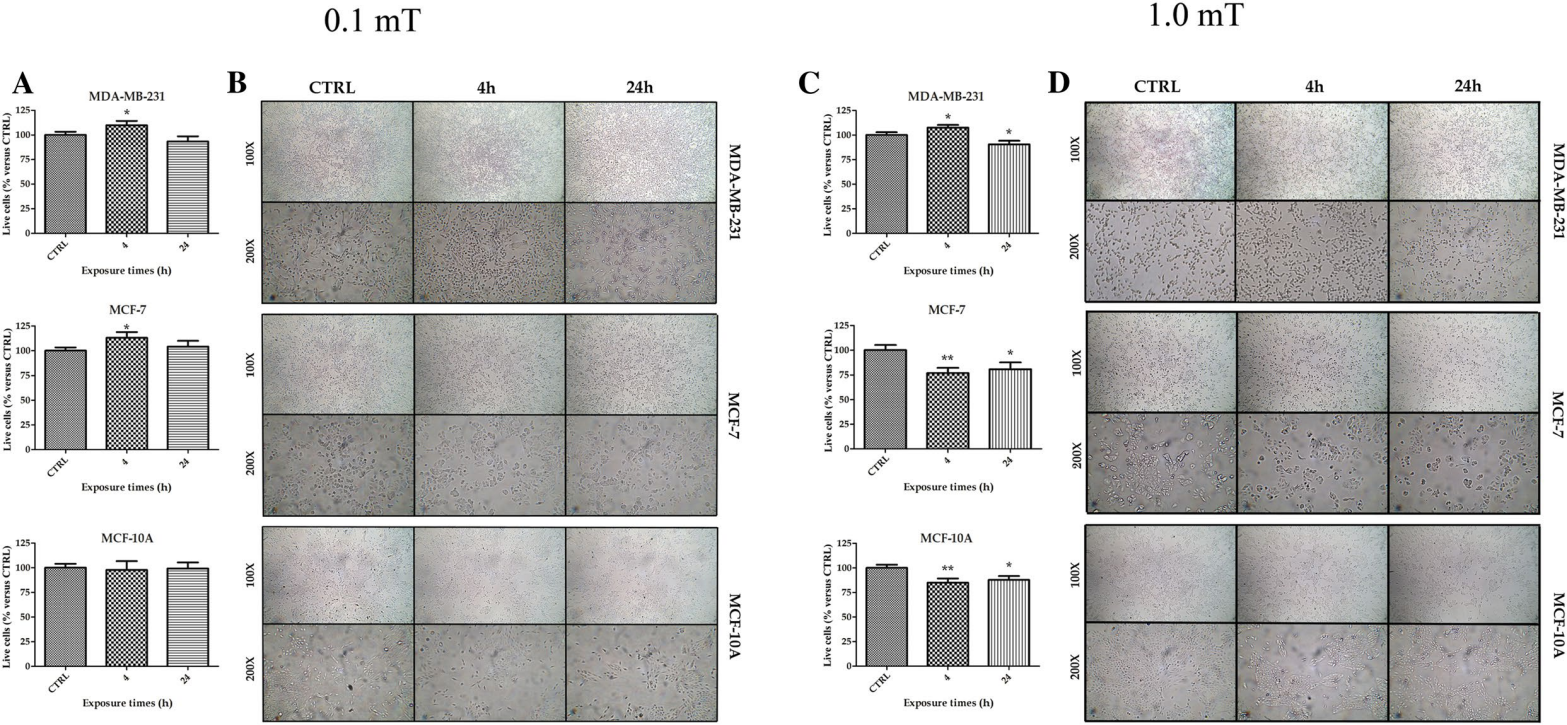
Number of live cells after 96 h from start of ELF-MF exposure. Trypan blue assay performed on MDA-MB-231 and MCF-7 breast cancer cells and MCF-10A breast cells exposed to 50 Hz 0.1 mT ELF-MF (**A**) or 50 Hz 1.0 mT ELF-MF (**C**). Data were analyzed at 96 h after start of treatment. Values for the exposed cells were expressed as the percentage of their respective controls. Results show mean ± SD. *p < 0.05 and **p < 0.01 vs. CTRL (0 h). Pictures of breast cells after exposure for 4 and 24 h to 50 Hz 0.1 mT ELF-MF (**B**) or 50 Hz 1.0 mT ELF-MF (**D**). Pictures were taken at × 100 and × 200 magnification 96 h after ELF-MF exposure

A trypan blue assay was performed to evaluate the number of live cells after exposure to 50 Hz 0.1 mT or 1.0 mT ELF-MF for 4 h and 24 h. Cells were counted 96 h after ELF-MF treatment. Both breast cancer cell lines, MDA-MB-231 and MCF-7 (Fig. 2A, B), showed an increase in the number of viable cells after exposure to 0.1 mT ELF-MF for 4 h compared with their respective unexposed controls (0 h). Conversely, we did not observe significant differences in MCF-10A live cell number after 0.1 mT ELF-MF cell exposure (Fig. 2A, B). In MDA-MB-231 cells, 1.0 mT ELF-MF exposure for 4 h induced an increase in the live cell number and a decrease after 24 h of exposure compared to control cells (Fig. 2C, D). Both MCF-7 and MCF-10A (Fig. 2C, D) breast cells exposed to 1.0 mT ELF-MF for 4 or 24 h showed a reduction in the number of cells compared to controls. Cells were investigated 192 h after start of 4 h of ELF-MF exposure (Supplementary file Fig. 2). The number of live cells among 0.1 mT ELF-MF-treated MDA-MB-231 breast cancer cells was increased compared with that in the corresponding controls, no differences were seen in MCF-7 and MCF-10A breast cells. The number of live MDA-MB-231 breast cancer cells exposed to 1.0 mT was higher compared with unexposed cells, no differences were seen in MCF-7. The number of live MCF-10A cells was decreased, compared to that in controls.

### Exposure to 50 Hz ELF-MF altered the cell cycle in MDA-MB-231 and MCF-10A breast cells

**Fig. 3:**
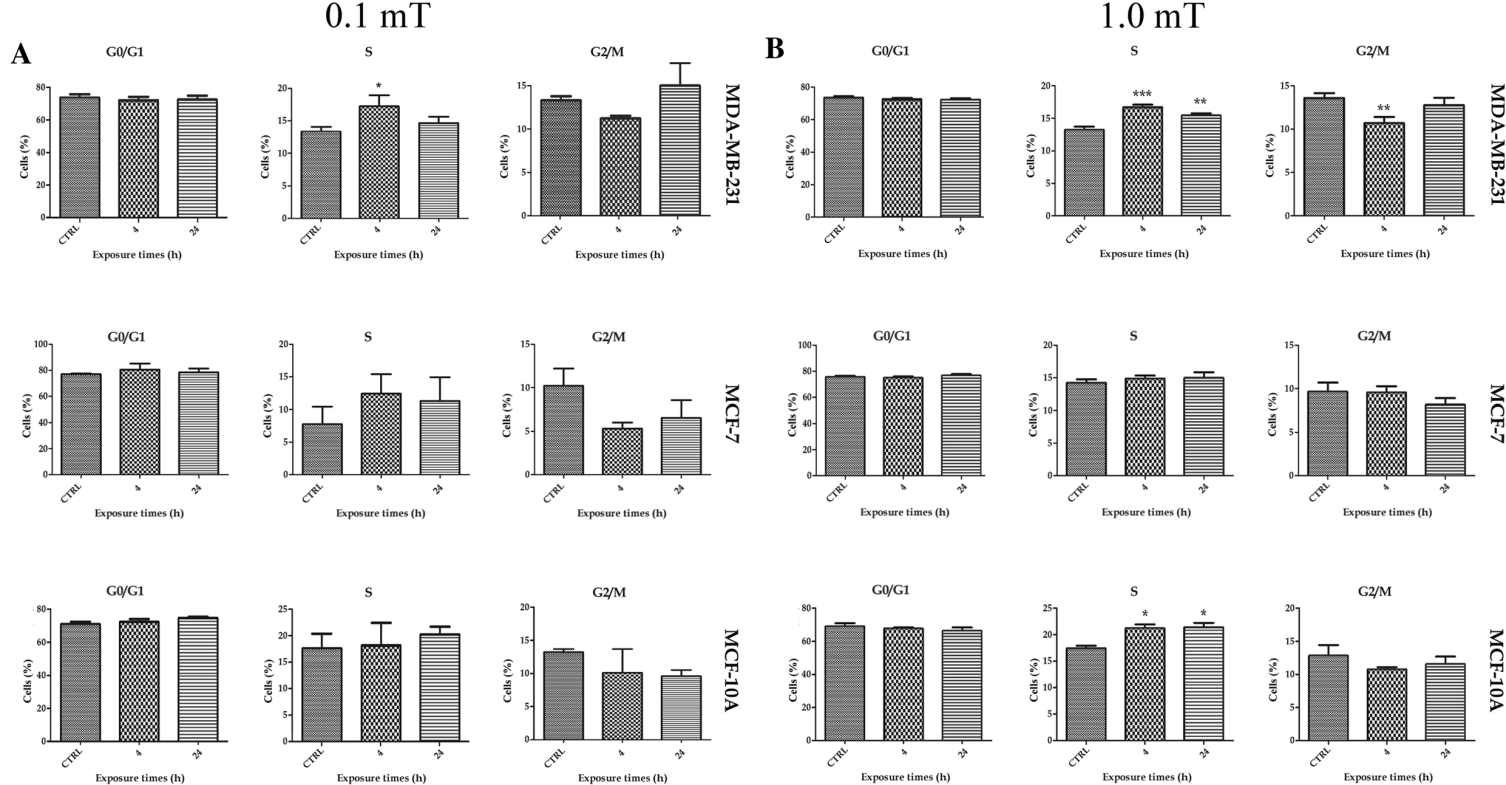
Cell cycle phases after exposure to 50 Hz ELF-MF. Cell cycle analysis after exposure to 50 Hz 0.1 mT ELF-MF (**A**) or 50 Hz 1.0 mT ELF-MF (**B**). Data were analyzed at 96 h after start of treatment. Results show mean ± SD. *p < 0.05, **p < 0.01 and ***p < 0.001 vs. CTRL (0 h)

The percentage of cells in each phase of the cell cycle was analyzed 96 h after 4 and 24 h of 50 Hz 0.1 mT ELF-MF or 1.0 mT ELF-MF exposure. For MDA-MB-231 cells, exposure to 0.1 mT ELF-MF for 4 h resulted in an increase in the number of cells in S phase compared with that in the control group (Fig. 3A). Cell cycle dynamics in the MCF-7 or MCF-10A cell lines were not modified after 0.1 mT ELF-MF treatment (Fig. 3A).

Exposure to 1.0 mT ELF-MF induced a significant increase in the number of cells in S phase among MDA-MB-231 cells treated for 4 h or 24 h and a significant reduction in the number of cells in G_2_/M phase in cells treated for 4 h compared to control cells (Fig. 3B). The MCF-7 cell line showed no changes in cell cycle phases (Fig. 3B). The analysis of cell cycle distribution in MCF-10A breast cells revealed an increased number of cells in S phase in response to 1.0 mT ELF-MF exposure for both 4 and 24 h (Fig. 3B).

### Exposure to 50 Hz ELF-MF promoted a change in the mitochondrial membrane potential (ΔΨ_M_) in breast cell lines

Cell viability is associated with mitochondrial activity; thus, we investigated whether 50 Hz ELF-MF could promote any changes in ΔΨ_M_ immediately after ELF-MF exposure for 4 h and 24 h. Moreover, to obtain data comparable to those from the previous analyses, we also investigated the ΔΨ_M_ in cells exposed for 4 h and 24 h and analyzed at 96 h from cell seeding.

**Fig. 4:**
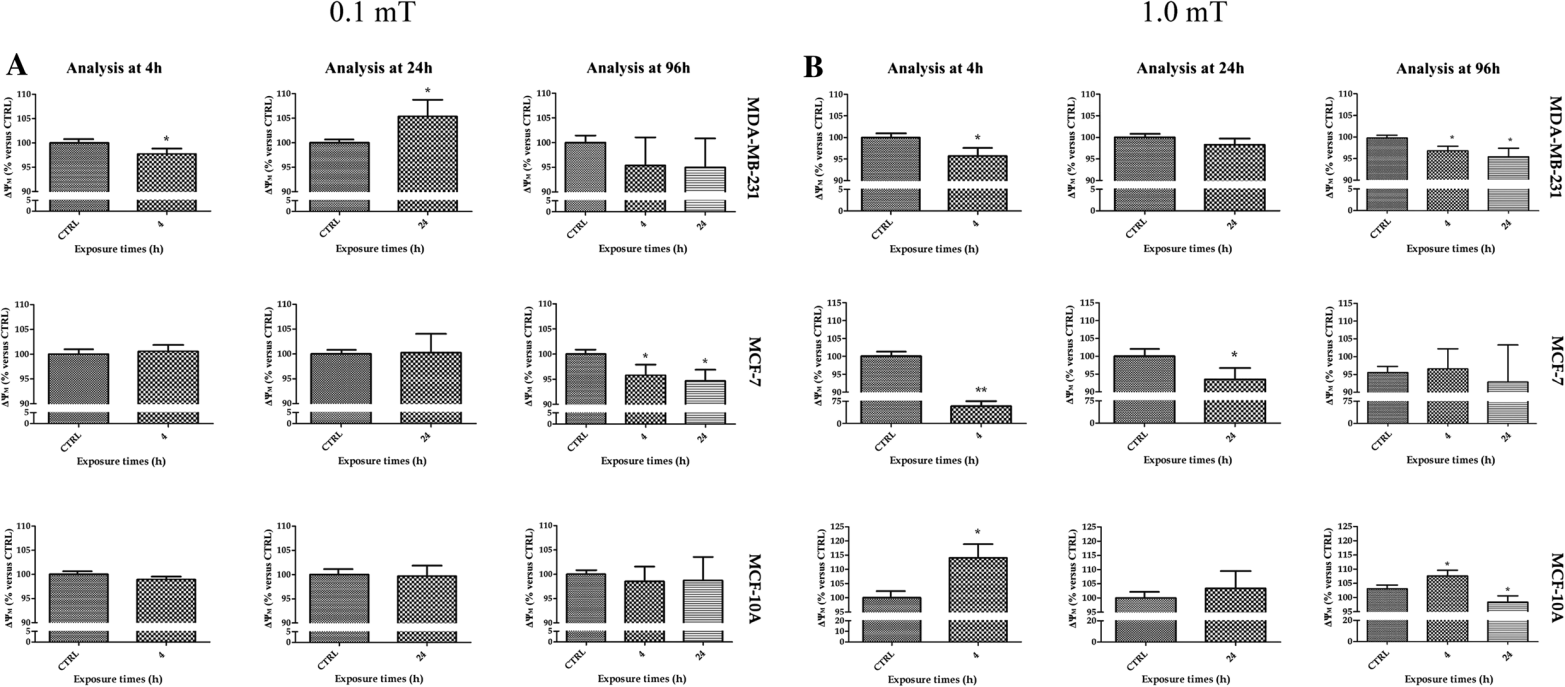
Effects of 50 Hz ELF-MF on mitochondrial membrane potential (ΔΨ_M_). 50 Hz 0.1 mT ELF-MF (**A**) or 50 Hz 1.0 mT ELF-MF (**B**) affect ΔΨ_M_ at different times. The ΔΨ_M_ was analyzed immediately after 4 h and 24 h of treatment, and 96 h after exposure to the ELF-MF. Values were expressed as the percentage of their respective controls. Data shown are mean ± SD. *p < 0.05 and **p < 0.01 vs. CTRL (0 h)

Triple-negative breast cancer cells showed lower and higher ΔΨ_M_ than unexposed cells immediately after 4 and 24 h, respectively, of exposure to 0.1 mT ELF-MF (Fig. 4A). Conversely, at 96 h after 0.1 mT ELF-MF treatment, no change in ΔΨ_M_ was observed (Fig. 4A). MCF-7 breast cancer cells showed a significant decrease in ΔΨ_M_ compared with control cells after 4 and 24 h of exposure only when ΔΨ_M_ was analyzed at 96 h (Fig. 4A). The ΔΨ_M_ values of MDA-MB-231 breast cancer cells were reduced when analyzed immediately after 4 h of 1.0 mT ELF-MF exposure. In addition, these cells showed a reduction in ΔΨ_M_ compared to unexposed cells when analyzed at 96 h from seeding after 4 and 24 h of exposure to 1.0 mT ELF-MF (Fig. 4B). MCF-7 breast cancer cells presented lower ΔΨ_M_ values immediately after both 4 and 24 h of 1.0 mT ELF-MF exposure (Fig. 4B). However, the differences were not statistically significant in these cells at 96 h after 1.0 mT ELF-MT exposure. MCF-10A breast cells showed an increase in ΔΨ_M_ values compared to control cells after 4 h of 1.0 mT ELF-MF exposure, and the difference persisted 96 h after exposure (Fig. 4B). A decrease in the ΔΨ_M_ value compared to that of the control was observed in cells treated with 1.0 mT ELF-MF for 24 h analyzed after 96 h (Fig. 4B).

### Exposure to 50 Hz ELF-MF promoted changes in ROS production in tumor breast cell lines

**Fig. 5:**
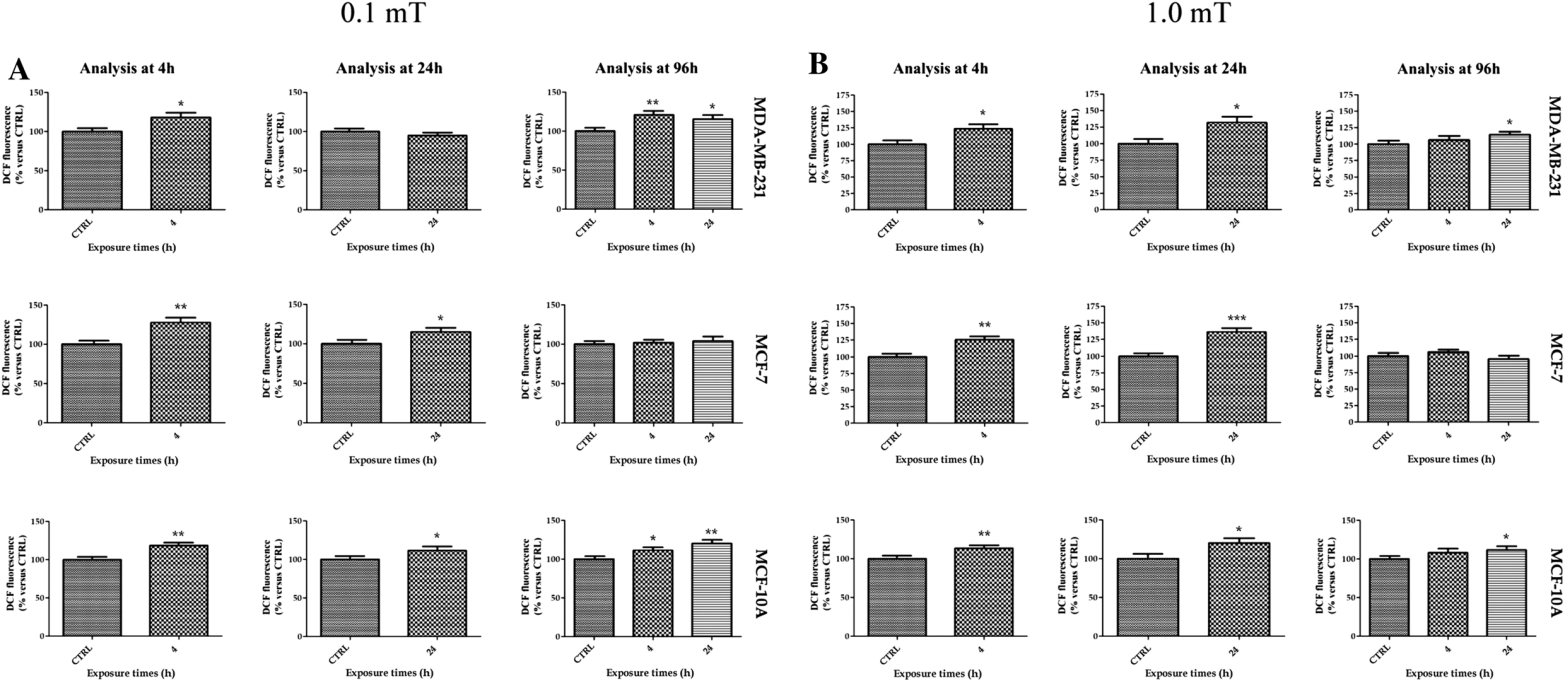
Exposure to 50 Hz ELF-MF altered ROS production in breast cells. ROS production was analyzed at 4 h, 24 h, and 96 h after exposure to 50 Hz 0.1 mT ELF-MF (**A**) or 50 Hz 1.0 mT ELF-MF (**B**). Values were expressed as the percentage of their respective controls. Data shown are mean ± SD. *p < 0.05, **p < 0.01 and ***p < 0.001 vs. CTRL (0 h)

MDA-MB-231 breast cells showed higher ROS levels than unexposed cells immediately after 4 h of 0.1 mT ELF-MF exposure and after 96 h of 4 or 24 h of 0.1 mT ELF-MF exposure (Fig. 5A). The ROS levels in MCF-7 cells were higher than those in unexposed cells immediately after 4 h and 24 h of 0.1 mT ELF-MF exposure. In contrast, no significant differences were observed after 96 h in MCF-7 breast cancer cells (Fig. 5A). Higher ROS levels were found in MCF-10A breast cells after 4 h and 24 h of 0.1 mT ELF-MF exposure than in unexposed cells. The same response was identified in MCF-10A cells exposed to 0.1 mT ELF-MF for 4 h or 24 h and analyzed after 96 h (Fig. 5A).

All three breast cell lines analyzed showed an increase in ROS levels compared to those in nonexposed cells after both 4 h and 24 h of 1.0 mT ELF-MF exposure (Fig. 5B). The ROS levels of MDA-MB-231 cells were increased in cells exposed to 1.0 mT ELF-MF and analyzed after 96 h (Fig. 5B). No significant changes in ROS levels were detected in MCF-7 cells analyzed 96 h after 1.0 mT ELF-MF exposure (Fig. 5B). For MCF-10A cells, higher ROS levels were observed in cells exposed to 1.0 mT ELF-MF for 24 h and analyzed after 96 h (Fig. 5B).

## Discussion

This study evaluated the influence of different exposure time (maximum 24 h) to 0.1 mT or 1.0 mT ELF-MF on viability of three breast cell lines, two of which are cancerous. Two ELF-MF exposure time (4 h and 24 h) were selected to study cellular proliferation, cell cycle distribution, ROS and mitochondrial membrane potential. The time of 4 h was investigated in order to replicate a plausible ELF-MF exposure in the workplace and 24 h was studied as the extreme condition of exposure.

The data obtained on viability did not show statistically significant differences with both ELF-MF flux densities on all three breast cell lines after 48 h from start of ELF-MF exposure. Our results are in line with studies that evaluated the viability of breast cells exposed to ELF-MF within 48 h of exposure [27, 30–32]. However, according to the results obtained by Lee et al. [33], the effects on the vitality and proliferation of breast cells may be evident after 3–4 days of exposure.

For this reason, the viability test was repeated 96 h after the start of exposure to ELF-MF. The data obtained indicate an influence of ELF-MF on the viability of all three cell lines related to the ELF-MF exposure time. The 0.1 mT ELF-MF exposure showed an increase in viability compared to the control in breast cancer cell lines. The increase in viability is associated with an increase in breast cancer live cells after 4 h exposure compared to the control. Exposure to 1.0 mT ELF-MF produces an increase in viability of breast cancer cells limited to short exposures, while in all three mammary cell lines there is a decrease in viability with an exposure of 24 h. The number of live MDA-MB-231 cells was increased in group exposed 1.0 mT ELF-MF for 4 h while was decreased in group exposed for 24 h. MCF-7 and MCF-10A cell lines showed a decrease in live cells following 4 h or 24 h 1.0 mT ELF-MF treatment. Our data confirm the findings of Lee et al. on MCF-10A cells exposed to 4 h of 1.0 mT ELF-MF and analyzed 96 h after exposure [33]. Our results suggest that the influence of ELF-MF exposure on proliferation may take time (> 48 h) to emerge and the effect is related to the exposure time. Few hours of ELF-MF exposure lead to an increase in viability of breast cancer cells after 96 h. This effect may be related an indirect action of ELF-MF on proliferation through the activation of molecular mediators which in turn increase the proliferation rate. After 192 h from few hours (4 h) of ELF-MF exposure no differences of cells viability were detected in all three ELF-MF treated breast cell lines. Differences in the number of live cells persisted after 192 h from ELF-MF exposure but they are similar to results observed after 96 h. Taken together these results suggest that the ELF-MF action on breast cells viability is time limited, it appears after several hours and then tends to decrease over time.

High intensity and longtime ELF-MF exposures (i.e. 1.0 mT, 24 h), on the other hand, lead to a decrease in proliferation in all three cell lines studied. An inhibitory effect on proliferation of ELF-MF on breast cells was observed in other studies after ELF-MF exposures higher than our treatments [22, 34]. The different effect of ELF-MF on cell proliferation probably is mediated by factors that stimulate the cells at low doses but are harmful at higher doses. In human neuroblastoma cells ELF-MF (0.1 mT 50 Hz) induces a proliferative response mediated by epithelial growth factor receptor (EGFR) and a subsequent activation of the mitogen-activated protein kinases (MAPKs) pathways [35]. The MAPKs signaling triggered by ELF-MF can explain both the stimulation and the inhibition of the breast cell viability. Indeed, MAPKs play a pivotal role in converting extracellular stimuli into a wide range of cellular responses and they have a dual role on cell fate since they can act as activators or inhibitors of apoptotic processes [36, 37].

The study of the cell cycle distribution of the three cell lines showed an influence of the ELF-MF exposure on cell cycle both in breast cancer and normal cells. An increase of cell number in S phase was obtained in MDA-MB-231 cells after 4 h of exposure to 0.1 mT ELF-MF, this increase was similarly obtained in the same cell line and in MCF-10A with exposure to 1.0 mT ELF- MF of 4 and 24 h. An effect of 1.0 mT 50 Hz ELF-MF on the cell cycle of mammary cells by an increase of cell number in S-phase cells was observed by Han et al. after an exposure to 1.0 mT 50 Hz ELF-MF of 12 h [30]. Since ELF-MF has been associated with altered mitochondrial activity [38, 39] and it can influence viability [3, 4], we assessed whether ELF-MF could promote changes in the mitochondrial membrane potential of breast cell lines. Results indicate that both ELF-MF flux densities studied were able to influence mitochondrial membrane potential. Stable mitochondrial membrane potential levels are required for mitochondrial and cellular health. Both prolonged state of depolarization or hyperpolarization results in mitochondria damage and cell apoptosis [40]. Several processes regulate mitochondrial membrane potential including oxidative phosphorylation and transport of charged compounds (e.g. Ca^2+^). ELF-MF may alter the mitochondrial membrane potential interfering both with oxidative phosphorylation and Ca^2+^ homeostasis [39, 41]. Our results showed that the action of ELF-MF exposures on mitochondrial membrane potential differs among breast cell lines and among ELF-MF exposure times. How ELF-MF produces mitochondrial membrane hyperpolarization or depolarization is probably related on the characteristics of ELF-MF exposure (e.g. duration and flux density) and cell type (e.g. metabolic needs and homeostatic capabilities). The mitochondrial membrane potential and the ROS production are strictly related, both hyper and depolarization of mitochondrial membrane can be associate with high ROS levels [40, 42]. The data obtained in this work related to the ROS production, both immediately after exposure to ELF-MF and after 96 h, showed that an exposure to ELF-MF can increase the production of ROS in breast cells and it depends on the cell line. The increase in ROS production is greater with the exposures to 1.0 mT ELF-MT supporting the association between ELF-MF exposure and oxidative status, at or above 1.0 mT [43].

ROS influence cell proliferation and their action depends on their amount. Notably, a small production of ROS can lead to an increase in cell proliferation while an excess of them would lead to cell damage and reduced proliferation [44, 45]. ROS production in turn can act as a potent regulator of MAPKs signaling cascade [46, 47]. A low intensity and/or short-term exposure to ELF-MF may lead to a slight increase in ROS levels that constitute a cellular proliferative stimulus if are mantained in a physiological range (e.g. they are counteracted by the cellular antioxidant systems) [46]. Conversely, a higher increase in ROS associated with higher ELF-MF intensity and longer exposure time may lead to a ROS ammount that if it exceeds a critical threshold the cellular oxidative damage prevails decreasing cellular proliferation [46]. The effect on ROS production may be present even after 96 h of exposure except in MCF-7 cells. The different behavior could also depend on the intrinsic characteristics of each breast cell line [48]. However, alternative pathways outside ROS production should be considered. A direct action of ELF-MF on EGFR and subsequently on MAPKs pathway may be supposed [35]. More, ELF-MF may act through effects on plasma membrane [49, 50], matrix metalloproteinases activity [19, 35], alteration of calcium signaling pathway [41] or alternative signaling pathways. Further studies to elucidate molecular pathways involved in breast cell response to ELF-MF exposure are needed. Our evidences support the Protection Guidelines Report of the ICNIRP where it is affirmed that the elucidation of the observed biological effects of ELF-MFs is complicated due to the lack of models explaining this phenomenon and the great variety of possible cell responses, depending on the type of cells, the exposure time, and the power density of the field [12].

Our data demonstrate that different magnetic flux densities but also different 50 Hz ELF-MF exposure times can have opposite effects on in vitro viability and proliferation of breast cells. Effects are visible after 96 h from exposure. The mitochondrion of breast cells is a target organelle for ELF-MF as well as the ROS production. Our data, although limited to in vitro exposures, stimulate further studies to investigate possible effects on mammary cells of ELF-MF exposures of magnetic flux densities compatible with the law limits for the general population and for people exposed in the workplaces.

## Supplementary Information

Below is the link to the electronic supplementary material.

Supplementary file1 (DOCX 413 kb)

## Data Availability

The data that support the findings of this study are available from the corresponding author upon reasonable request.
