# Supplementary material for: A 50 Hz magnetic field influences the viability of breast cancer cells 96 h after exposure
Source: Mol Biol Rep. 2022 Nov 15;50(2):1005–17. doi: 10.1007/s11033-022-08069-7 (PMC9889515; doi:10.1007/s11033-022-08069-7)
Supplement: Supplementary file 1 — Supplementary file1 (DOCX 413 kb) [file 11033_2022_8069_MOESM1_ESM.docx]

### SUPPLEMENTARY MATERIAL


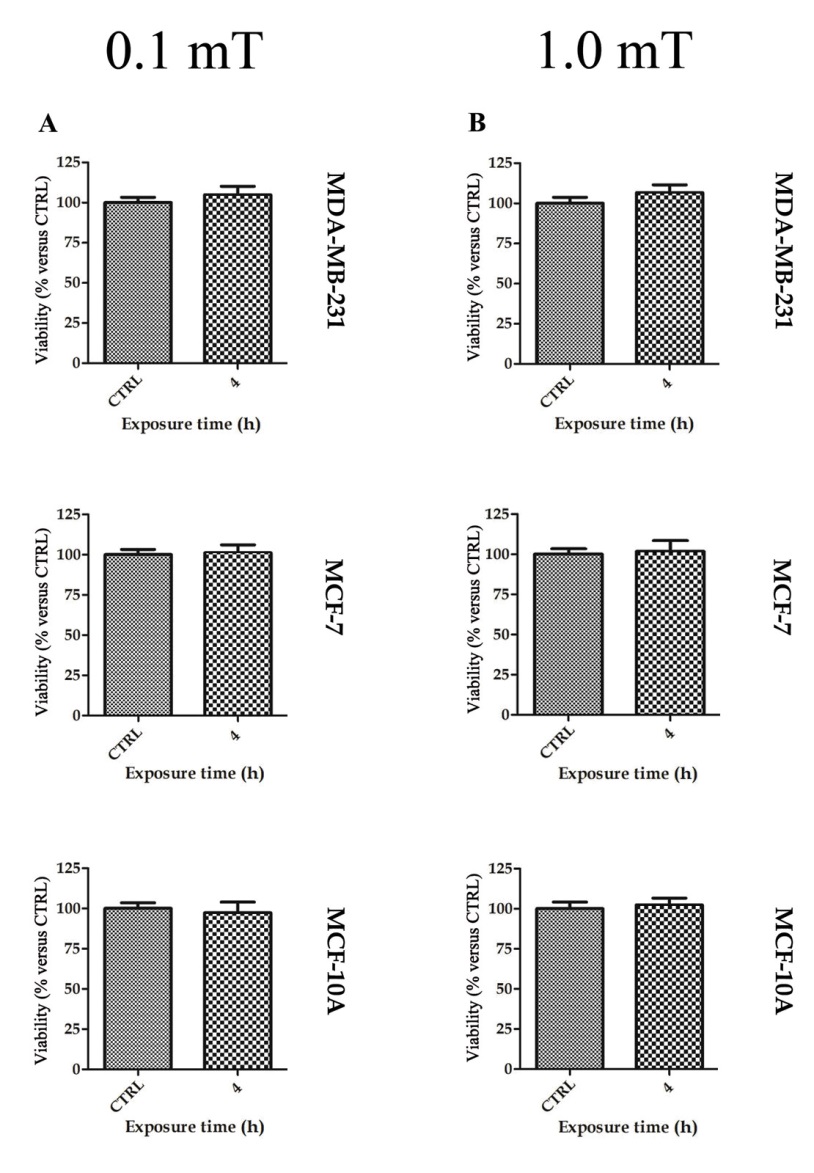


**Supplementary file, Fig. 1 Cell viability after 192 h from the 50 Hz ELF-MF exposure at 0.1 and 1.0 mT of MB-MDA-231, MCF-7 and MCF-10A cell lines.** Cells were exposed to 50 Hz 0.1 mT ELF-MF (A) or 1.0 mT ELF-MF (B) for 4 h and the viability was analyzed through an XTT assay at 192 h after start of treatment. Values for the exposed cells were expressed as the percentage with respect to the not exposed group. Data show mean ± SD


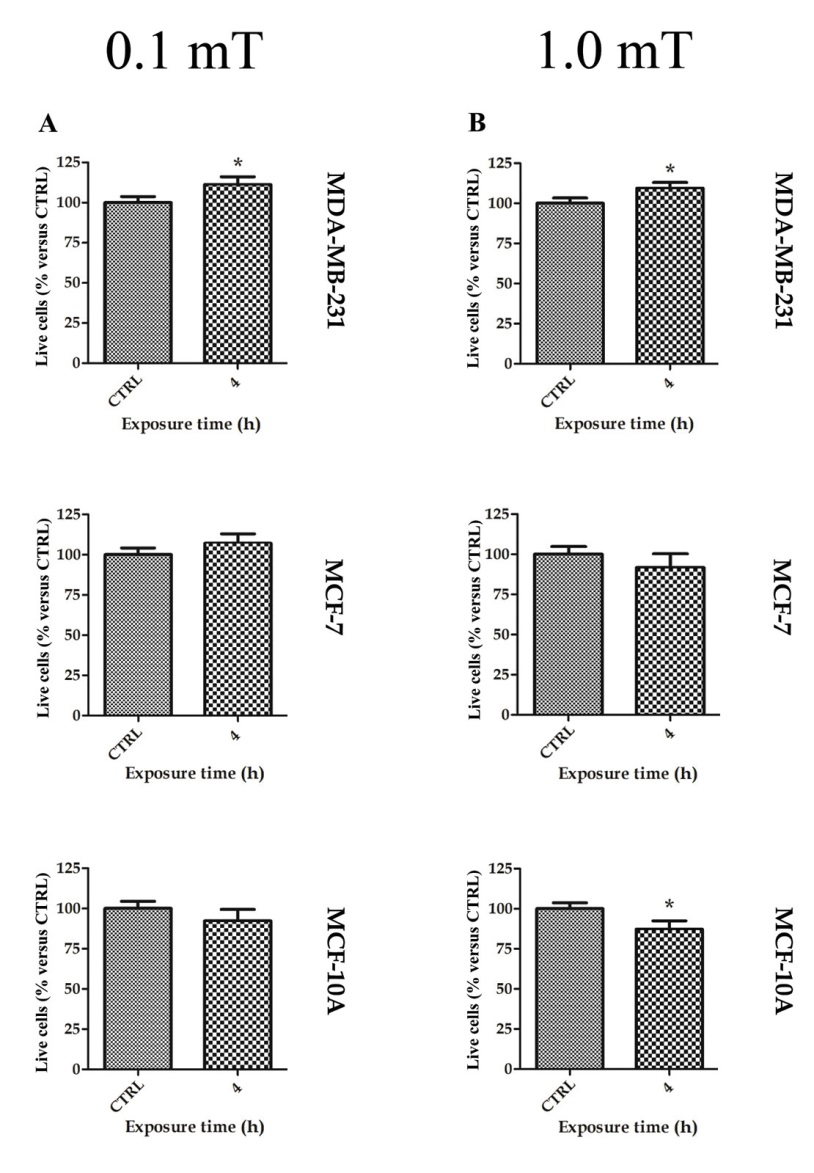


**Supplementary file, Fig. 2 Number of live cells after 192 h from start of ELF-MF exposure.** Trypan blue assay performed on MDA-MB-231 and MCF-7 breast cancer cells and MCF-10A breast cells exposed to 50 Hz 0.1 mT ELF-MF (A) or 50 Hz 1.0 mT ELF-MF (B). Data were analyzed at 192 h after start of treatment. Values for the exposed cells were expressed as the percentage of their respective controls. Results show mean ± SD. * = p<0.05 vs. CTRL (0 h)
